# P02.106. Eurhythmy Therapy in the aftercare of children and adolescents with brain tumors of the posterior fossa: a pilot study

**DOI:** 10.1186/1472-6882-12-S1-P162

**Published:** 2012-06-12

**Authors:** J Kanitz, K Pretzer, P Hernaiz Driever, G Calaminus, A Wiener, G Henze, G Seifert

**Affiliations:** 1 Charité, University of Medicine, Berlin, Germany; 2 University Hospital of Muenster, Pediatric and Adolescent Medicine, Muenster, Germany; 3 University of Muenster, Pediatric and Adolescent Medicine, Muenster, Germany

## Purpose

The side effects and sequelae related to anti-tumor therapy regularly result in extensive physical, psychosocial and mental impairments in the life of children after therapy of a brain tumor. Although some supportive therapies are available, there are still numerous unsolved chronic problems. Therefore innovative additional therapeutic approaches from the field of complementary medicine should also be evaluated. This pilot study is a first orienting attempt to assess the feasibility, treatment adherence and impact of Eurythmy Therapy (EYT) in pediatric oncology. EYT is a movement therapy that belongs to the field of Mind-Body Therapies (MBTs). This holistic approach aims to promote self regulation and self-healing powers, e.g. in cancer patients.

## Methods

This paper addresses results of seven patients who participated in 25 sessions of EYT over six months with a follow-up period. The outcome parameters, cognitive functioning, neuromotor functioning and visuomotor integration, were assessed prior to the beginning of the intervention and after 6 and 12 months.

## Results

First results show feasibility and excellent adherence and indicate positive improvements in cognitive and neuromotor functioning in all children and better visuomotor integration in five out of seven children after six months. After 12 months, neuromotor functioning and visuomotor integration had diminished again to some extent.

## Conclusion

The current findings suggest that children with cancer affecting the central nervous system may profit from EYT.

